# Management of a giant aortic root aneurysm in a young patient with Marfan syndrome: a case report

**DOI:** 10.1186/s13019-020-01304-x

**Published:** 2020-09-24

**Authors:** Jiayu Shen, Changping Gan, R. D. T. Rajaguru, Dou Yuan, Zhenghua Xiao

**Affiliations:** Department of Cardiovascular Surgery, West China Hospital of Sichuan University, No.37 Guo Xue Alley, Chengdu, 610041 Sichuan China

**Keywords:** Aortic aneurysm, Cardiac surgery, Dilated aortic root, Marfan syndrome

## Abstract

**Introduction:**

Marfan syndrome (MFS) is a common heritable connective tissue disease involving multiple organs. Even though the clinical manifestations of MFS can be various, aortic root aneurysm is estimated as one of the most serious complications. We herein describe an individualized treatment decision-making process for a 23-year-old male with MFS, suffering from a giant but stable aortic root aneurysm which is extremely rare at his age.

**Case:**

The patient, a 23-year-old male with a family history of MFS, presented to our cardiovascular department because of progressive exertional chest distress, fatigue and occasional precordial pain. Physical examinations revealed 190.5 cm of height, high myopia, and a diastolic murmur at the aortic valve area. Laboratory examinations for systemic vasculitis and infectious diseases were negative. Transthoracic echocardiography and enhanced thoracic computed tomography (CT) scan revealed the existence of a giant aortic root aneurysm (*125.1 mm in short-axis*), severe aortic valve regurgitation, cardiac dilatation (*LV; 99 mm in diastolic diameter*) and a poor ejection fraction (*EF; 18%*). Considering the risk of rupture or dissection of the dilated aortic root, we performed Bentall procedure based on the results of multidisciplinary team discussion and intraoperative exploration. Postoperative thoracic CT scan revealed a normal sized reconstructed aortic root, and the patient was discharged uneventfully 7 days later.

**Conclusion:**

It is extremely rare to report such a giant aortic root aneurysm in a young patient. In the treatment decision-making process, the patient’s specific situation should be taken into consideration. A mechanical Bentall procedure seems to be an acceptable option for some selected cases.

## Introduction

Marfan syndrome (MFS) is an autosomal dominant disorder of connective tissues, which is primarily associated with the mutation of FBN-1 gene on chromosome 15q21 encoding fibrillin-1, an essential glycoprotein in the extracellular matrix [1]. Aortic root aneurysm and ectopia lentis are considered as the cardinal features of MFS [2]. Indeed, it is the progressive dilation of the aorta leading to aortic rupture or dissection that affects the prognosis. Meanwhile, aortic regurgitation secondary to aortic root dilation leads to ventricular volume overload, left ventricular dilation and eventually cardiac function impairment [3]. In order to reduce the risk of aortic dissection or secondary cardiac dysfunction, replacement of the dilated aortic root with a valved conduit has remained as the mainstay treatment strategy, whereas it may be met with anticoagulation-related complications and/or limited durability [4]. In recent years, with the deepening understanding of the anatomical structure of the aortic root and the increasing regard to long-term prognosis, valve-sparing operations have become a promising treatment strategy for patients suffering from aortic root aneurysm. Its application, however, is still restricted in some specific conditions, such as the significantly dilated aortic annulus and severely damaged aortic valve [5]. We herein present our experience in managing a young MFS patient with a giant aortic root aneurysm by tailoring an individualized surgical regimen on the premise of a comprehensive perioperative assessment.

## Case

**Fig. 1:**
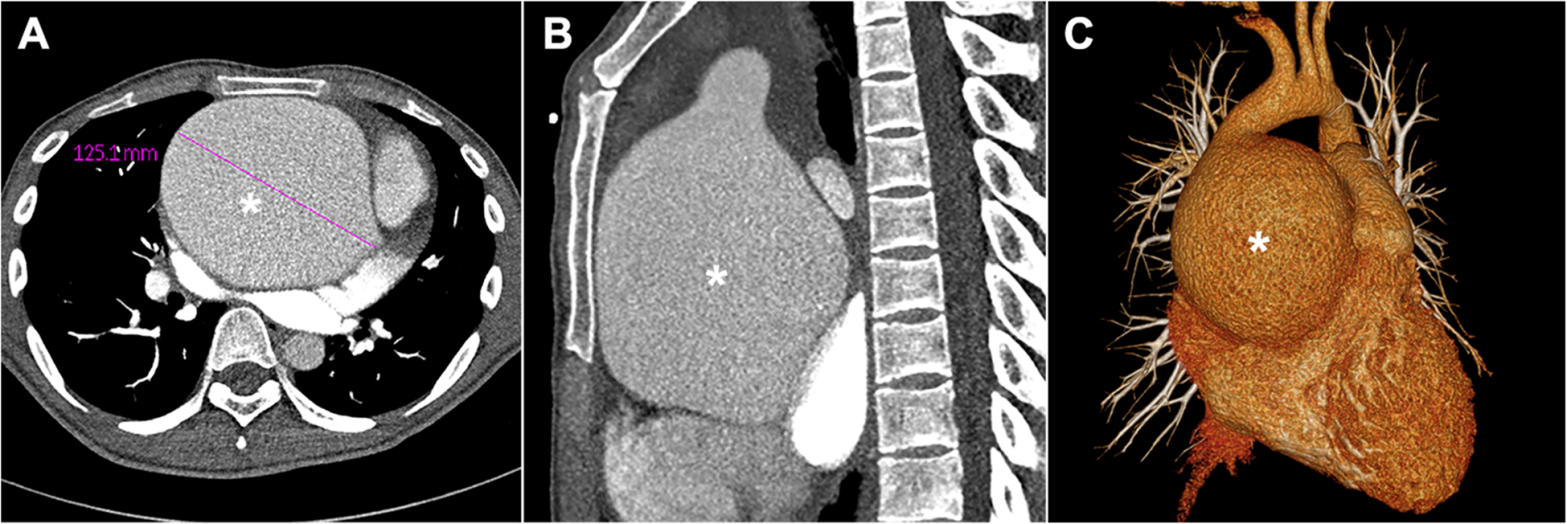
Results of preoperative imaging examinations: **a** and **b** Transverse view and sagittal view of enhanced thoracic computed tomography scan demonstrated the size of the aneurysm, respectively; **c** Three-dimensional enhanced computed tomography scan showed that the aortic root aneurysm is like a “bulging balloon”; *, aortic root aneurysm

A 23-year-old male presented to our cardiovascular department with progressive exertional chest distress and fatigue for over one year. One month before the admission, the patient experienced novo occasional precordial pain in addition to the presenting symptoms. On admission, physical examinations revealed heart rate of 96 beats/minute with a sinus rhythm, blood pressure of 130/55 mmHg, high myopia, 190.5 cm of height and a diastolic murmur at the aortic valve area. Laboratory examinations for systemic vasculitis and infectious diseases were unremarkable. Transthoracic echocardiography (TTE) demonstrated a giant aortic root aneurysm, an enlarged left ventricle (*LV; 99 mm in end-diastolic dimension*) and severe aortic regurgitation combined with extremely poor ejection fraction (*EF; 18%*). No mitral regurgitation or tricuspid regurgitation were observed. Enhanced thoracic CT scan performed with a 16-detector row confirmed the extensive dilated aortic root aneurysm, bulging against the sternum (*Fig. **1**a and b, asterisks; 125.1 mm in short-axis*), while the morphology of the distal of ascending aorta and descending aorta were normal (*30 mm in short-axis*). Three-dimensional enhanced CT scan revealed that the aortic root aneurysm is like a “bulging balloon” (*Fig. **1**c, asterisk*). According to the patient’s self-statement, both of his grandpa and father died from aortic dissection caused by MFS, and he was identified with *FBN1* mutations when he was a child. Unfortunately, the patient was lost to follow up owing to his poor compliance. Taken together, this patient was diagnosed with MFS based on the Revised Ghent Criteria [2].

**Fig. 2:**
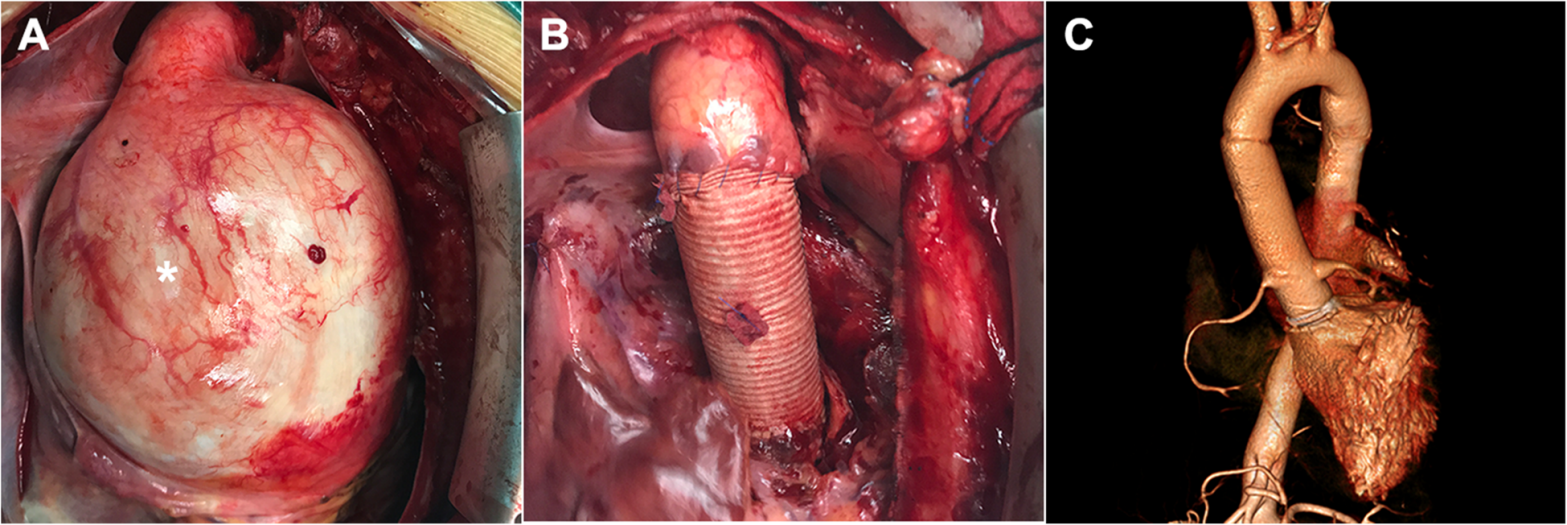
Results of intraoperative exploration and postoperative imaging examination: **a** The aortic root aneurysm compressed the right atrial and the pulmonary artery; **b** The aortic root aneurysm was removed after Bentall procedure; **c** Three-dimensional enhanced computed tomography scan of pre-discharge showed a normal sized aortic root

The cardiac dysfunction of this patient was improved after receiving cardiotonic and diuretic therapy. After multidisciplinary discussion, surgical intervention, instead of continuously medical therapy, was accepted as reasonable and life-saving, in consideration of the risk of rupture/dissection of the aortic root aneurysm and deteriorating cardiac function. The specific surgical approaches should be decided based on the intraoperative exploration results. Elective femoral arteriovenous cardiopulmonary bypass was performed to avoid unexpected rupture of the giant aneurysm during median sternotomy. Following pericardiotomy, the aneurysm almost completely filled the pericardial cavity and compressed the right atrial and the pulmonary artery (*Fig. **2**a, asterisk*). After clamping the aorta and opening the aneurysm sac, we observed a tricuspid aortic valve with multiple fenestrations occurring towards the commissures, a torn leaflet and a dilated aortic annulus which was measured over 38 mm. Further exploration verified the morphology of mitral valve was normal. Considering the patient’s poor cardiac function with bad aortic valvular condition and he may not be able to tolerate time-consuming procedures, we decided to perform Bentall procedure with a 28-mm conduit composite a 25-mm mechanical aortic valve (*Fig. **2**b*). Postoperative three-dimensional enhanced CT scan revealed a normal sized reconstructed aortic root (*Fig. **2**c*) and pre-discharge TEE examination confirmed the patient’s EF was improved to 40% and the LV end-diastolic dimension was reduced to 59 mm. The patient received anticoagulation therapy and he was discharged uneventfully 7 days later. Regular follow up was required to reduce the risk of anticoagulation-related complications. Moreover, enhanced thoracic CT scan and echocardiography examination were required at least once per year to monitor the morphology of valved conduit and the cardiac function and valvular condition of this patient.

## Discussion

MFS is a common autosomal dominant genetic disorder caused by *FBN1* mutations [6]. The reported prevalence varies from 1 to 3 per 10,000 individuals, depending on the diagnostic criteria and ethnicity, while no sex predilection is apparent in MFS [7, 8]. Although this monogenic connective tissue disorder generally segregates as a dominant trait in families, novo mutations are responsible for nearly 25% of cases [9]. Even though MFS affects various systems including skeletal, oclar and cardiovascular systems, the latest nosology emphasizes on the alterations in the cardiovascular system which is the most detrimental phenotype [2]. Indeed, dissection or rupture of the dilated aortic root which is seen in nearly three quarters of patients with MFS is the predominant cause of mortality in these cases, with a peak incidence in the third and fourth decades of life [3, 6, 9].

Usually, MFS patients are asymptomatic when the dilated aorta remains stable. However, the dilation rate of the aorta is heterogeneous and cannot be predicted. Although the risk of type A dissection clearly increases with the increasing aortic root diameter, it can also occur even in patients with mild aortic dilation [5], just like an unstable deadly “bulging balloon”. Moreover, if the dilation involves the aortic annulus and aortic valve is anatomically changed, cardiac dysfunction secondary to valvular regurgitation would be the dominant clinical feature. In this patient, even though the aortic root aneurysm has dilated to such rare giant scale, it remained stable and the clinical symptoms were mainly due to cardiac dysfunction secondary to aortic regurgitation and an enlarged left ventricle.

Early screening and establishment of the diagnosis of aortic aneurysm in patients with a family history of MFS is critical since prophylactic intervention can reduce the risk of aortic dissection and rupture effectively, which requires effective screening methods. As most of the early stage patients are asymptomatic, the aortic aneurysm is usually found occasionally during a routine X-ray, which shows a widened mediastinum [4]. Furthermore, echocardiographic assessment can provide us with detailed information regarding the morphological features of the left ventricle, aortic annulus, aortic sinuses and ascending aorta in multiple views and dynamic videos in evaluating aortic valve morphology and the mechanism of aortic insufficiency [10, 11]. Due to its convenience and safety, echocardiography has been widely used in young individuals who require repetitive imaging and long-term follow-up. However, echocardiographic evaluation alone cannot offer information on adjacent structures or the involvement of aortic branches. In this case, furthermore, we performed an enhanced thoracic CT scan to visualize the main aortic branches and measure the extent and size of the aneurysm accurately.

Once the diagnosis of aortic aneurysm has been confirmed in patients with MFS, intense physical activity such as weightlifting should be avoided due to the potential risk inducing rupture or dissection of the aortic aneurysm [12] Meanwhile, regular follow-up is necessary. Concurrently, conservative management is aimed at decreasing the heart rate and lowering the blood pressure to reduce the hemodynamic stress on the proximal aorta [13]. β-adrenergic receptor antagonists, including propranolol, atenolol and nebivolol [14–16] have become the most prescribed medications for MFS patients as they might decrease the aortic dilation rate [17]. Losartan, one of the angiotensin II receptor 1 blockers (ARBs), is useful in inhibiting the dilation of the aorta as it was recently discovered that angiotensin II is involved in MFS pathophysiological process [18]. However, more clinical trials are required to verify the ability of ARBs to interfere with the MFS pathology. Even though several novel therapeutic strategies are under investigation, the goal of precision medicine is laborious to achieve [19]. Surgical intervention remains as the gold standard treatment strategy for aneurysm in MFS.

Surgical treatment should be considered in MFS patients who have aortic root dilatation with a maximal diameter ≥ 50 mm [20]. If there are additional risk factors including family history of aortic dissection, severe aortic regurgitation, desire for pregnancy, systemic hypertension and/or aortic size increase > 3 mm/year, surgical intervention is recommended when maximal aortic diameter ≥ 45 mm [20]. Bentall procedure (composite replacement of the aortic valve and dilated ascending aorta combined with coronary artery reimplantation) has become a low-risk and durable operation with 5- and 10-year survival rates of 84% and 75%, respectively [21]. Meanwhile, aortic valve-sparing operations such as David’s procedure (reimplantation of the aortic valve) and Yacoub’s procedure (remodeling of the aortic root) in young patients with aortic aneurysm have represented promising treatment strategies in recent years [22]. Even though Yacoub’s procedure has been reported to be inappropriate for patients with Marfan syndrome because of the significant aortic insufficiency and reoperation rate [23], David’s procedure shows excellent outcomes [24]. In this case, the significant dilation of the aortic annulus caused structural damage to the aortic cusps and multiple fenestrations were detected around commissures. While David’s procedure with an external annuloplasty band can correct the dilated aortic annulus, this outcome may not last for long [25]. More importantly, in this case, our patient is not a suitable candidate for time-consuming valve-sparing procedure in considering his poor cardiac function. As the body surface area of this patient was measured as 1.99m^2^ and the morphology of the distal of ascending aorta was normal, we finally decided to perform Bentall procedure for this patient with a 28-mm conduit composite a 25-mm mechanical aortic valve in order to balance the risk of prosthesis-patient mismatch and the suitability of grafted conduit.

Based on our limited experience learned from this case, we suggested that the management options for MFS patients with aortic root aneurysm should depend on the valvular anatomical morphology and patients’ overall health status. For patients with anatomically normal valve combined with acceptable cardiac function, valve-sparing operation should be attempted as it reduces the risk of thromboembolism, hemorrhage and infective endocarditis, especially in young patients. Otherwise, Bentall procedure should be performed, and lifelong anticoagulation therapy is mandatory in young patients.

## Conclusion

The treatment decision-making process should depend on the patients’ specific situations. Our practice indicates that the mechanical Bentall procedure remains a valuable approach in some selected patients.

## Data Availability

All data generated or analyzed during this study are included in this published article.

## Abbreviations

CT: Computed tomography
EF: : Ejection fraction
MFS: : Marfan syndrome
TTE: : Transthoracic echocardiography
ARBs: : Angiotensin II receptor 1 blockers
